# Sustainment, Sustainability, and Spread Study (SSaSSy): protocol for a study of factors that contribute to the sustainment, sustainability, and spread of practice changes introduced through an evidence-based quality-improvement intervention in Canadian nursing homes

**DOI:** 10.1186/s13012-019-0959-2

**Published:** 2019-12-19

**Authors:** Whitney B. Berta, Adrian Wagg, Lisa Cranley, Malcolm B. Doupe, Liane Ginsburg, Matthias Hoben, Lauren MacEachern, Stephanie Chamberlain, Fiona Clement, Adam Easterbrook, Janice M. Keefe, Jennifer Knopp-Sihota, Tim Rappon, Colin Reid, Yuting Song, Carole A. Estabrooks

**Affiliations:** 1 Institute of Health Policy, Management & Evaluation, University of Toronto, Dalla Lana School of Public Health, 155 College Street, Suite 425, Toronto, Ontario M5T 3M6 Canada; 2 Division of Geriatric Medicine, Department of Medicine, Faculty of Medicine & Dentistry, University of Alberta, 1-198 Clinical Sciences Building, 11350 - 83 Avenue, Edmonton, Alberta T6G 2P4 Canada; 3 Lawrence S. Bloomberg Faculty of Nursing, University of Toronto, 155 College Street - Suite 130, Toronto, Ontario M5T 1P8 Canada; 4 Departments of Community Health Sciences and Emergency Medicine, Manitoba Centre for Health Policy, Manitoba Training Program for Health Services Research, Max Rady College of Medicine, Rady Faculty of Health Sciences, University of Manitoba, 408-727 McDermot Avenue, Winnipeg, Manitoba R3E 3P5 Canada; 5 School of Health Policy & Management, Faculty of Health, York University, HNES 413, Toronto, Ontario Canada; 6 Faculty of Nursing, University of Alberta, 5-305 Edmonton Clinic Health Academy (ECHA), 11405 87 Avenue, Edmonton, Alberta T6G 1C9 Canada; 7 Department of Family Medicine, University of Alberta, Alzheimer Society of Canada Postdoctoral Fellow, 6-50 University Terrace, University of Alberta, Edmonton, Alberta T6G 2T4 Canada; 8 O’Brien Institute for Public Health, Cumming School of Medicine, University of Calgary, 3rd Floor Training Research and Wellness Building, 3280 Hospital Drive NW, Calgary, Alberta T2N 4Z6 Canada; 9 Centre for Health Evaluation and Outcome Sciences (CHÉOS), St. Paulʼs Hospital, 588–1081 Burrard Street, Vancouver, British Columbia, V6Z 1Y6 Canada; 10 Nova Scotia Centre on Aging, Department of Family Studies and Gerontology, Mount Saint Vincent University, Halifax, Nova Scotia BEM 2J6 Canada; 11 Faculty of Health Disciplines, Athabasca University, 6th Floor, South Campus, 345 – 6 Avenue SE, Calgary, Alberta T2G 4V1 Canada; 12 School of Health and Exercise Sciences, Faculty of Health and Social Development, University of British Columbia – Okanagan, 1147 Research Road, Kelowna, British Columbia V1V 1V7 Canada; 13 Translating Research in Elder Care (TREC), Faculty of Nursing, University of Alberta, 5-007D Edmonton Clinic Health Academy (ECHA), 11405 87 Avenue, Edmonton, Alberta T6G 1C9 Canada; 14 Faculty of Nursing, University of Alberta, 5-183, Edmonton Clinic Health Academy, 11405 87 Ave, Edmonton, Alberta T6G 1C9 Canada

**Keywords:** Long-term care, Nursing homes, Sustainability, Quality improvement, Evidence-based care practice

## Abstract

**Background:**

Implementation scientists and practitioners, alike, recognize the importance of sustaining practice change, however post-implementation studies of interventions are rare. This is a protocol for the Sustainment, Sustainability and Spread Study (SSaSSy). The purpose of this study is to contribute to knowledge on the sustainment (sustained use), sustainability (sustained benefits), and spread of evidence-based practice innovations in health care. Specifically, this is a *post-implementation* study of an evidence-informed, Care Aide-led, facilitation-based quality-improvement intervention called SCOPE (*Safer Care for Older Persons (in long-term care) Environments*). SCOPE has been implemented in nursing homes in the Canadian Provinces of Manitoba (MB), Alberta (AB) and British Columbia (BC). Our study has three aims: (i) to determine the role that adaptation/contextualization plays in sustainment, sustainability and spread of the SCOPE intervention; (ii) to study the relative effects on sustainment, sustainability and intra-organizational spread of high-intensity and low-intensity post-implementation “boosters”, and a “no booster” condition, and (iii) to compare the relative costs and impacts of each booster condition.

**Methods/design:**

SSaSSy is a two-phase mixed methods study. The overarching design is convergent, with qualitative and quantitative data collected over a similar timeframe in each of the two phases, analyzed independently, then merged for analysis and interpretation. Phase 1 is a pilot involving up to 7 units in 7 MB nursing homes in which SCOPE was piloted in 2016 to 2017, in preparation for phase 2. Phase 2 will comprise a quasi-experiment with two treatment groups of low- and high-intensity post-implementation “boosters”, and an untreated control group (no booster), using pretests and post-tests of the dependent variables relating to sustained care and management practices, and resident outcomes. Phase 2 will involve 31 trial sites in BC (17 units) and AB (14 units) nursing homes, where the SCOPE trial concluded in May 2019.

**Discussion:**

This project stands to advance understanding of the factors that influence the sustainment of practice changes introduced through evidence-informed practice change interventions, and their associated sustainability. Findings will inform our understanding of the nature of the relationship of fidelity and adaptation to sustainment and sustainability, and afford insights into factors that influence the intra-organizational spread of practice changes introduced through complex interventions.

Contributions to the literature

- Post-implementation studies of intervention sustainability, like that described in this protocol, are rare.
- Once intervention implementation supports are removed, the effects of interventions are susceptible to natural decay and our understanding of how to sustain the use of the new knowledge conveyed in interventions, post-implementation and long-term, is poor.
- This protocol describes a study that will contribute to knowledge on the sustainment (sustained use), sustainability (sustained benefits), and spread of evidence-based practice innovations in health care.
- The study will be situated in the under-studied institutional long-term care sector.

## Background

Considerable investment is made to generate research knowledge intended to improve the quality and delivery of health care. Knowledge of this type, particularly when it is complex, is frequently conveyed via evidence-based practice interventions, and the costs expended to implement these interventions are similarly substantial [1]. Once intervention implementation “supports” are removed, the initial effects obtained through these interventions are susceptible to natural decay [2–4]. The long-term durable sustainment of evidence-based changes to practice is challenging [1, 5, 6].

While sustainability has been identified as “one of the most significant translational research problems of our time” (1: 2), post-implementation studies of practice change sustainability in health care are rare [7–11] and so it follows that our understanding of the factors that influence sustainability is generally poor. Failure to sustain evidence-based changes or innovations to practice means that the intended improvements to care are short-lived, that the practice innovations’ further scale-up and spread is unlikely, and that real losses are incurred on research investment, often made with public funds. *This protocol describes a study that aims to contribute to our understanding of the inter-related phenomena of sustainability, sustainment, and spread of evidence-informed, complex practice change interventions*.

### Unpacking the concept of sustainability

While the concept of sustainability is still maturing [3, 4, 12], work in this area has recently acknowledged a useful distinction between sustainability and sustainment [4, 8]. With a focus on lasting benefits, *sustainability* generally refers to the extent to which “an evidence-based intervention can deliver its intended benefits over an extended period of time after external support from the donor agency is terminated” ([13]: 118); whereas, *sustainment* refers to the continued enactment of processes, practices, or work routines that are conveyed and learned through an intervention [4, 8]. While the concept of spread is generally discussed separately [14], we suggest that there is likely a link between spread and the concepts of sustainability and sustainment, given that the spread of the practices and benefits introduced through an intervention, from one part of an organization to other parts or from one organization to another, is unlikely to take place without some degree of retention of these processes and benefits within the originating unit or organization. As with sustainability, the importance of understanding the processes and factors that influence the spread of healthcare innovations, including practice innovations, are highlighted in the implementation literature, albeit separate from the literature on sustainability [9, 15, 16].

### Approaches to studying sustainability: fidelity versus adaptation

To date, studies of sustainment and sustainability invoke one of two dominant and competing approaches: the fidelity approach, and the adaptation approach [3, 7]. The *fidelity approach* focuses on implementation fidelity and is the most common approach used to examine sustainability. Fidelity is defined as the extent to which an intervention program follows the originally intended implementation plan and faithfully delivers the research-informed components of the intervention [11, 17]. This approach contends that deviation from the intended intervention content and delivery protocols during implementation—that is, “program drift” and “low fidelity”—will inevitably lead to diminished benefits/outcomes both during and after implementation, once intervention support is withdrawn [8, 18].

By contrast, the *adaptive approach* ascribes importance to the co-evolution of the intervention and the organizational context into which it is introduced [7]. This approach suggests that overemphasis on fidelity and adherence, “relative to generalizability and adaptation”, increases the risk of creating interventions—including practice changes and the processes to effect them—that will not “fit” within complex or changeable settings ([8]: 2), and that while changes to the intervention may reduce fidelity they may lead to *improved* fit to local context and *enhanced* outcomes/benefits [8, 9, 18, 19].

In this study, fidelity versus adaptation is of interest to us to the extent that it is, or is not, related to post-implementation sustainment and sustainability of practice change. High implementation fidelity *during* an intervention may contribute to sustained use and benefits. Conversely, the adaptive perspective suggests that sustainability and sustainment is achieved in organizations that are adept at striking a balance between fidelity and responsiveness to the implementation context. The bottom line is that what is done during implementation, in addition to what is done afterwards, is thought to affect the sustainment, sustainability and spread of practice changes conveyed through an intervention—but we do not know precisely how. Work to further our understanding of relationships amongst fidelity, adaptation, sustainment, sustainability, and spread is needed and there is almost no literature on these dynamics. SSaSSy will contribute to this understanding.

### Study context

SSaSSy is a post-implementation study of sustainment (continued use), sustainability (lasting benefits), and spread (beyond the initial implementation setting) of the practice changes conveyed through an evidence-informed, Care Aide-led, facilitation-based quality-improvement intervention called SCOPE (*Safer Care for Older Persons (in long-term care) Environments*) that is the focus of a clinical trial being conducted in in Canadian nursing homes operating in the Provinces of Manitoba (MB), Alberta (AB) and British Columbia (BC) [NCT03426072]. In SCOPE, HCA-led teams lead quality-improvement initiatives focussing on one of three resident care areas identified as priorities by experts in long-term care, i.e., mobility, pain, and behavior [20]. The SCOPE intervention was piloted in nursing homes in AB and BC over 2010-2011. The impacts of the SCOPE intervention are described in several published articles:

- In Norton et al. [21], the SCOPE pilot was shown to meet its primary objective of demonstrating the feasibility and utility of implementing the intervention in nursing homes relying upon the leadership of HCAs, and engagement of professional staff and leadership in facilitative roles. Specifically, of the 10 HCA-led QI teams in nursing homes that participated in the SCOPE pilot, 7 succeeded in learning and applying the improvement model and methods for local measurement, with 5 of the 10 teams showing measurable improvement in the chosen clinical areas.
- These impacts were corroborated in a follow-up study that examined the sustainability of elements of the SCOPE pilot [22]. In this article, sustained differences between participating/intervention units, and non-participating units, were observed in outcomes relating to quality-improvement activities (i.e., continuation of work according to the improvement model and principles learned in SCOPE), HCA empowerment, and satisfaction with quality of work life.
- As part of the SCOPE clinical trial, SCOPE was implemented in 7 units in MB nursing homes over 2017, somewhat earlier than the intervention was implementation in participating BC and AB units/homes. While the data for the MB homes will be analyzed in conjunction with that collected for homes in BC and AB, a recent retrospective qualitative analysis of the implementation experiences [23] of administrative leaders (sponsors), professional staff, and QI team participants in MB homes demonstrates effects akin to those observed in the SCOPE pilot. In addition to accruing observable benefits to residents who were the subjects of the QI initiatives formulated by the HCA-led QI teams in participating units in each MB home, SCOPE was observed to change the expectations and behaviors on the part of administrative leaders, professional staff, and—importantly—HCAs relating to their abilities to conduct small-scale, unit-level, evidence-informed quality-improvement initiatives [23].

Both SCOPE and SSaSSy are situated within a larger program of research, Translating Research in Elder Care (TREC) [24]. TREC was initiated in 2007 and focuses on the influence of organizational context on resident quality of care and safety in 94 nursing homes in the three Western Canadian Provinces [24]. Both SCOPE and SSaSSy rely on TREC’s longitudinal database that includes data on staff behavior, attitudes and quality of worklife; leader behavior; work environment (context); and care unit and nursing home characteristics (e.g., unit size, facility owner-operator model). Data are collected routinely from all levels of nursing home staff, and quality of care measures are collected on a quarterly basis across the 94 homes at the unit level [25] via the Resident Assessment Instrument—Minimum Data Set, version 2.0 (RAI-MDS 2.0).

### Study purpose and aims

SSaSSy focusses on a 1-year interval, 1 year *after* SCOPE implementation concludes. Phase 1 of SSaSSy is a pilot that will occur in 7 units in MB nursing homes where SCOPE was piloted over 2016–2017. The results of this pilot will inform the content of post-implementation “boosters”, designed to sustain or renew the application of the QI techniques and tools—the changes to care practice—conveyed through SCOPE. The relative effectiveness of the boosters compared to a no booster control will be tested in phase 2 in 31 more units in nursing homes in BC and AB, where SCOPE concluded in May 2019.

As a post-implementation study, SSaSSy presents a rare opportunity to systematically contribute to knowledge [22] on the sustainment and sustainability of complex practice changes conveyed through evidence-based interventions, and to examine spread—first, in the SCOPE pilot sites in MB and subsequently in the trial sites in BC and AB.

Specific aims of SSaSSy are:

1. To determine whether fidelity, site- or facility-initiated adaptation of aspects of the intervention, aspects of the implementing unit, and/or other aspects of nursing homes’ operations or structures, are associated with sustainment, sustainability and spread one year following implementation of practice changes conveyed through SCOPE.
2. To explore the relative effects on sustainment, sustainability and intra-organizational spread of high- and low-intensity post-implementation “boosters” compared to “no booster”/natural decay; specifically, the extent to which there are:
  - sustained or renewed improvements in resident outcomes in clinical areas of focus targeted by the SCOPE intervention (deteriorating mobility, pain, responsive behavior) (*sustainability*),
  - sustained or renewed changes in staff behaviors (reported use of best practices, use of SCOPE components and processes) (*sustainment*),
  - sustained or renewed changes to staff work attitudes (work engagement, psychological empowerment, burnout, job satisfaction) and outcomes (organizational citizenship behaviors) related to work performance (*sustainability*),
  - sustained or renewed changes to senior leadership support behaviors relating to staff engagement in SCOPE (*sustainment*),
  - indications of *spread* to other units within the SCOPE intervention sites, and its extent.
3. To compare the costs and effectiveness of each post-implementation support condition.

## Approach/methods

SSaSSy is a two-phase mixed methods study. The overarching design is convergent, with qualitative and quantitative data collected over a similar timeframe in each of the two phases, analyzed independently, then merged, interpreted and reported by means of joint display [26].

### Participating nursing home sites

SSaSSy will first take place in the nursing home units in MB that participated in the SCOPE pilot, and then in those in AB and BC that participated in the full SCOPE trial. These homes meet the inclusion criteria applied to the original SCOPE pilot, and trial: (i) the facility provides 24-h on-site housing and health care services care for older adults by professional (nursing) staff and others; (ii) the facility is registered with the provincial government; (iii) 90% of residents are aged 65 or over; (iv) RAI-MDS 2.0 has implemented since January 2011; (v) facility operations are conducted in the English language; (vi) urban facilities are located within designated health regions and within 110 km of the TREC-designated hub for the health region.

### Phase 1

The first study phase, in MB, entails developing the content of the two post-implementation support conditions—low- and high-intensity “boosters”—through consultation with participants in the SCOPE pilot sites in MB nursing homes; piloting data collection instruments with SCOPE pilot site participants that explore factors relating to sustainability, sustainment and spread including fidelity and adaptation relevant to aim 1; piloting the low- and high-intensity “boosters” in up to 7 SCOPE pilot sites in MB while collecting quantitative and qualitative data relevant to aim 2 (sustainability, sustainment, spread); and piloting a data collection instrument intended to capture the costs related to each of the post-implementation support conditions.

#### SSaSSy booster content

The preliminary content of the low- and high-intensity boosters is informed by three prior studies relating to the SCOPE intervention [22, 23, 27] that highlight four components of the SCOPE pilot that appear to be highly relevant to its implementation: (i) the presence of team and senior sponsors who learn leadership skills intended for use in supporting the Care Aide-led unit QI Teams by securing resources; (ii) face-to-face “Learning Congresses” in which Teams build QI-related skills and where exchanges with care teams from other facilities enhance learning and team efficacy; (iii) quality advisors who fill both supportive and educational roles relating to applying QI techniques on the part of the QI Teams, and change management leadership coaching of sponsors; and (iv) the use of “setting aims” as an effective mechanism for changing QI Team members’ behavior. The mix of these booster components, and their intensities, will be further informed through a focus group consultation of 2–3 decision-makers familiar with the MB SCOPE pilot, 2–3 QI experts, and 2 researchers with expertise in implementation science.

#### SSaSSy booster pilot

The low- and high-intensity boosters will be piloted in up to 7 units in 7 MB homes (potentially 3 low-intensity booster units, 4 high-intensity booster units) for 7 months, starting June 2019. The pilot interval will be followed by data analysis and refinement of boosters where we will focus on: (i) assessing relationships between the boosters’ contents and sustainment, sustainability, and spread; (ii) the clarity of the booster content from the perspective of the QI Teams; and (iii) the adequacy of the modes of delivery. Quantitative and qualitative data will be collected during the pilot; see Table 1 for a summary of measures relevant to each study aim.

**Table 1:** SSaSSy aims, measures and analyses

Aim		Data/measures	Analysis
Aim 1. Explore site adaptation and/or contextualization		Semi-structured in-person interviews with the QI Team lead, senior sponsor, and QI advisor	A hybrid approach of qualitative methods will be used, thematic analysis [28], which incorporates both an inductive approach that allows themes to emerge from the data [29] and a deductive a priori template-of-codes approach [30] based on research we review above. Audiotapes of the interviews will be transcribed verbatim and data analysis will be completed using line-by-line coding and constant comparative methods [31]
Aim 2: Explore the relative effects of the high- and low-intensity boosters	2a: Sustainability: Sustained or renewed improvements to quality of resident care	Unit-level quality indicators generated using RAI-MDS 2.0 data on resident outcomes (mobility, pain, behavior)	Run charts will be generated for the RAI-MDS data^(i)^ collected SSaSSy start for each of the three quality indicators for each unit (RAI data elements MOB01/MOB1A; PAI0X/PAN01; BEHD4/BEHI4). For the clinical area on which QI Teams focus, we will analyze the relevant RAI-MDS quality indicator using statistical process control methods [32] and a procedure that members of our research team developed for the SCOPE proof of principle study [21] which categorizes control charts in terms of demonstrated changes to performance.
Notes:			
(i) RAI-MDS 2.0 is a valid, reliable standardized assessment of resident outcomes that includes a comprehensive set of clinical outcomes and captures characteristics of nursing home residents and their care [33]. Each resident has a full assessment (~450 items) performed on admission, and a shorter (300 item) assessment is performed quarterly. These data are routinely collected from all 94 participating TREC sites, from which SCOPE study sites were drawn, and are the source of SSaSSy’s resident outcome data			
	2b & 2d: Sustainment: Sustained or renewed changes in staff behavior and senior leadership support behaviors	SCOPE templates completed by QI Team leads. diaries and feedback reports completed by QI Advisors	SCOPE Templates^(ii)^ will be analyzed, using document analysis [34, 35], at two junctures: analysis of the SCOPE Templates accrued over SCOPE implementation, and over the SSaSSy booster interval to determine peri-intervention fidelity and post-intervention fidelity, respectively. First, gross-grained assessments of the use/non-use of the templates (sustainment) by each QI Team will be made (i.e., were templates complete and posted on intranet site available to Teams). Thematic analysis (see above) will then be used to analyze the contents of the QI Team templates, as well as the QI advisor diaries and feedback reports^(iii)^
	2c: Sustainability: Sustained or renewed changes to staff work attitudes and outcomes related to work performance	TREC HCA Survey [36]	While quantitative data will be collected and analyzed over the course of the project, SSaSSy likely will not be adequately powered for statistical inference. Descriptive statistics including statistics of central tendency, dispersion, and standard deviation will be computed for each unit-level variable, for each booster arm collected through the TREC Survey waves. However, if changes in the primary outcome of the SCOPE intervention, Care Aide-reported *conceptual use of best practices*, are sufficient over the booster interval (e.g., an effect size of 0.29), we will use one-way ANOVA (repeated measures, within-between interaction) to test for “pre–post” differences in the means of each variable within and between groups/booster arms, followed by Tukey–Kramer test for significant differences between all pairs of groups if appropriate (where distributions are not normal the Kruskal–Wallis test will be used, and where data are heteroscedastic Welch's ANOVA will be used). In the more likely event that changes of this magnitude do not occur, we will compute p-values by time point before and after the SSaSSy booster interval. We are constrained in our sample size because we are studying the post-implementation of a trial with a fixed number of experimental sites.
(ii) SCOPE Templates refer to documents introduced during SCOPE implementation, in the all-team Learning Congresses, that are designed to assist care aide-led QI Teams in planning and managing their QI projects, and measuring and reporting their progress against their project aims			
(iii) The quality advisors keep diaries in which they prepared structured summaries of each interaction with the QI Teams, whether face-to-face or telephone, outside of the all-team Learning Congresses. Quality advisors also prepare written, structured, quarterly feedback reports for each QI Team			
	2e: Spread: Indications of spread to other units within the SCOPE intervention sites	Semi-structured in-person interview with the QI Team lead, senior sponsor, and QI advisor	As above
Aim 3: Compare the effectiveness and costs of post-implementation support arms		Documentation of resources and associated costs for low- and high-booster conditions	This cost analysis is exploratory in nature. We plan a simple, disaggregated reporting of costs. We will report the intervention costs (costs to deliver the boosters) separately from the costs incurred by the participating units. Each unit will report their incurred costs for staff, training and materials and supplies. Disaggregated presentation will allow units to understand what is driving the costs and identify possible areas where costs could be modified within the overall intervention

### Phase 2

The second study phase will use a quasi-experimental design [37] to evaluate the relative effectiveness of the three post-implementation support conditions: two treatment groups (low-, and high-intensity post-implementation boosters) and an untreated control group (no booster). Pretest and post-test data [37] relating to sustainment and sustainability will be collected through TREC surveys and via unit-level RAI-MDS 2.0 quality indicators. Phase 2 will begin in June 2020 and involve trial sites in BC (17 units) and AB (14 units), where SCOPE implementation concluded in May 2019. Specifically, the untreated control group (10 units) will receive no post-implementation support, one treatment group (10 units) will be provided with a low-intensity booster, and the second treatment group (11 units) will receive a high-intensity booster.

#### Assignment to treatment and control groups

We plan to use a cut-off-based random assignment strategy [37]. First, the extent to which SCOPE-conveyed practices have been sustained just prior to SSaSSy start-up in BC and AB, nursing homes will be assessed through “baseline interviews” with the team and senior sponsors. This will be followed by random assignment of those with high levels of sustained activity, and those with low levels, to each of the low-intensity booster, high-intensity booster, and untreated control groups. This approach will be piloted in phase 1 amongst participating units in MB nursing homes.

#### Inclusion of non-equivalent dependent variables for each group

The quasi-experiment design will be further strengthened by including non-equivalent dependent variables, in addition to the target outcomes variables. We have a ready way in which to do this: each QI Team is instructed to focus on one of either resident mobility, pain management or reducing responsive behaviors, and we collect RAI-MDS 2.0 quality indicator data (see Table 1, aim 2a) on each of these clinical areas. For a QI Team focussing on mobility, for example, we consider the measure based on mobility indicators MOB01 and MOB1A to be our target outcome variable, while PAI0X/PAN01; and BEHD4/BEHI4 will serve as our non-equivalent dependent variables. That is, while in this example neither the pain nor behavior measures would be predicted to change because of the treatment (SSaSSy), they would be expected to respond similarly to contextually important internal validity threats in the same way as the mobility target outcome.

### Measures and analysis

The relationships among the study aims, measures and analyses are summarized in Table 1.

### Integration

**Fig. 1:**
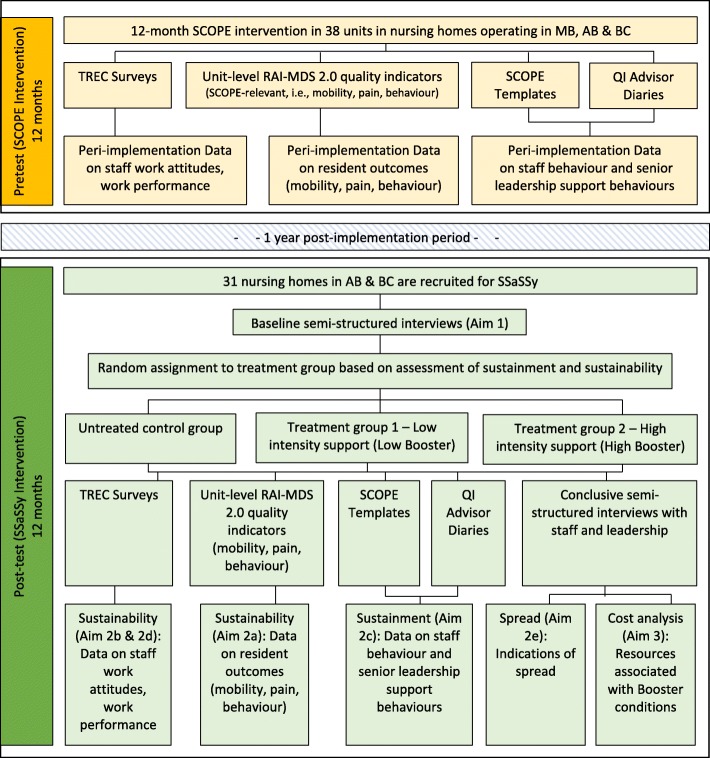
Phase 2 flow diagram

In this mixed methods study, quantitative and qualitative findings will be integrated at the interpretation and reporting stage [26, 38]. Independent analyses of the qualitative and quantitative data will serve to organize the data in a format based on thematic relevance (sustainability, sustainment, spread, influencing factors) that permit merging and subsequent higher order integration. This will be accomplished in two ways: first, the quantitative and qualitative data will be integrated using a joint display. Second, a narrative approach will be used to describe the quantitative and qualitative results thematically. The narrative will offer intra-group and inter-group comparisons within and between booster arms. Figure 1 offers a flow diagram for phase 2.

## Discussion

This study was developed in response to calls for studies that advance our understanding of the phenomena of post-implementation sustainability and sustainment of knowledge conveyed through evidence-based interventions [7, 8]. Failure to sustain evidence-based innovations to practice means that the intended improvements to care are short-lived, and that often considerable investments of health human resources are forfeit. We will also examine the phenomena of spread, as it seems reasonable to expect spread to be associated with sustainability and/or sustainment.

### Strengths and limitations

A significant strength of this study is that it relies upon multiple methods and multiple and diverse sources of data, with the survey and indicator data relying upon well-established, validated instruments with good psychometric properties. The quasi-experiment in phase 2 is strengthened by: a cut-off-based random assignment of units to treatment and untreated control groups; pretest and post-test measures; the inclusion of non-equivalent dependent variables for each of the three groups; and the inclusion of two comparison treatment groups and an untreated control group.

We are constrained in our sample size, because we are studying the post-implementation of a trial with a fixed number of experimental sites and therefore will not be adequately powered for statistical inference and must rely on descriptive statistics to examine the relative effectiveness of the booster and no booster control groups in phase 2.

## Conclusion

This project stands to advance the state of the science about factors that influence the sustained use (sustainment) of practice changes conveyed through interventions, and the associated sustained benefits of those changes to resident and staff outcomes (sustainability). Our findings will also inform discussion of the relevance of fidelity and adaptation to sustainment and sustainability, and offer insights into the factors that influence intra-organizational spread of complex interventions [39]. Finally, we will gain insights into the relative effects of differing intensities of post-implementation boosters vs. a no-booster untreated group, on the sustainment, sustainability and intra-organizational spread of practice changes introduced through SCOPE, in addition to the relative costs of these booster treatments. Importantly, SSaSSy focusses on the long-term effectiveness and sustainability of an intervention applied to long-term care settings, where post-implementation phenomena have not been studied, and where there is increasing concern for the costs, quality and sustainability of older adult care [40].

## Data Availability

Not applicable.

## Abbreviations

AB: Alberta
BC: British Columbia
HCA: Health care aid
MB: Manitoba
SCOPE: Safer Care for Older Persons (in long-term care) Environments
SSaSSy: Sustainment, Sustainability, and Spread Study
TREC: Translating Research in Elder Care
